# Spinal Projection Neurons Control Turning Behaviors in Zebrafish

**DOI:** 10.1016/j.cub.2013.06.044

**Published:** 2013-08-19

**Authors:** Kuo-Hua Huang, Misha B. Ahrens, Timothy W. Dunn, Florian Engert

**Affiliations:** 1 Program in Neuroscience, Harvard Medical School, 220 Longwood Avenue, Boston, MA 02115, USA; 2 Department of Molecular and Cellular Biology, Harvard University, 16 Divinity Avenue, Cambridge, MA 02138, USA

## Abstract

Discrete populations of brainstem spinal projection neurons (SPNs) have been shown to exhibit behavior-specific responses during locomotion [1–9], suggesting that separate descending pathways, each dedicated to a specific behavior, control locomotion. In an alternative model, a large variety of motor outputs could be generated from different combinations of a small number of basic motor pathways. We examined this possibility by studying the precise role of ventromedially located hindbrain SPNs (vSPNs) in generating turning behaviors. We found that unilateral laser ablation of vSPNs reduces the tail deflection and cycle period specifically during the first undulation cycle of a swim bout, whereas later tail movements are unaffected. This holds true during phototaxic [10], optomotor [11], dark-flash-induced [12], and spontaneous turns [13], suggesting a universal role of these neurons in controlling turning behaviors. Importantly, we found that the ablation not only abolishes turns but also results in a dramatic increase in the number of forward swims, suggesting that these neurons transform forward swims into turns by introducing turning kinematics into a basic motor pattern of symmetric tail undulations. Finally, we show that vSPN activity is direction specific and graded by turning angle. Together, these results provide a clear example of how a specific motor pattern can be transformed into different behavioral events by the graded activation of a small set of SPNs.

## Highlights

• Spinal projection neurons serve as a switch to transform forward swims into turns
• Visually induced and spontaneously occurring turns are controlled by the same set of SPNs
• Turn-controlling SPN activity is direction specific and graded by turning angle

## Results

### Detailed Tail Kinematics of Turning and Forward Swims

**Figure 1:**
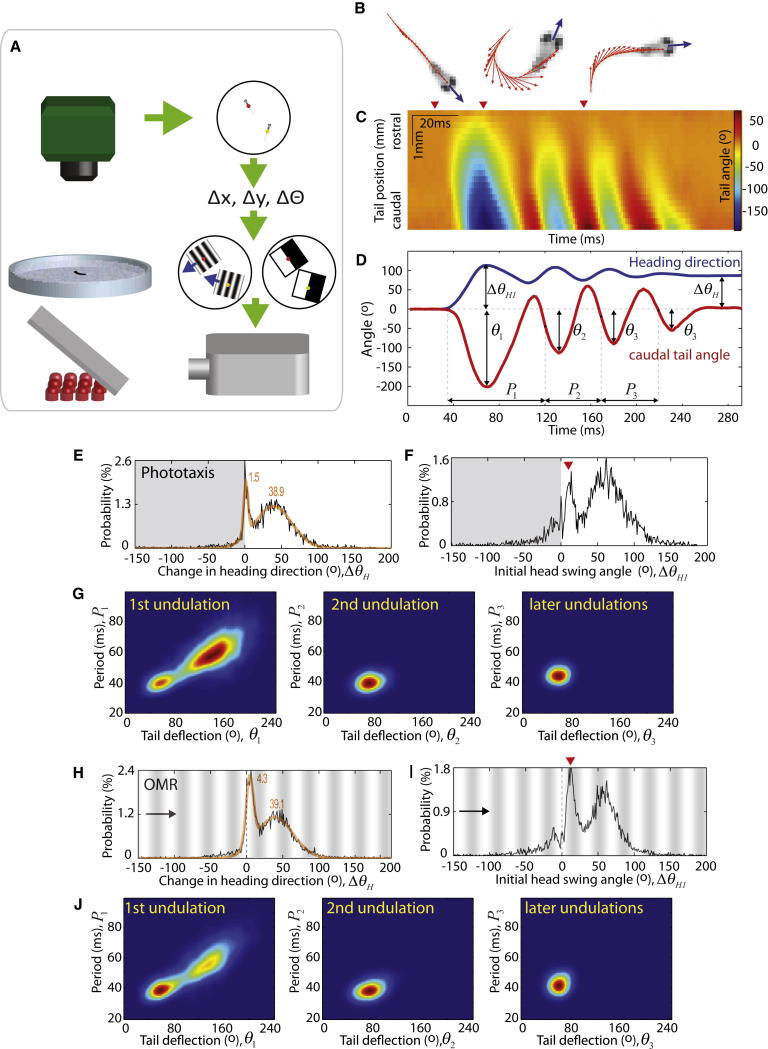
Detailed Swim Kinematics during Phototaxis and the Optomotor Response (A) Schematic of the behavioral setup. (B) Examples of the fish-tracking algorithm. Heading direction is indicated by the blue vector, and the tail shape is described by a series of tangent vectors (orange) along the tail. (C) Evolution of the tail shape during a turn. The angle differences between the heading direction and tail tangents are color coded in each column. (D) Undulations of the heading direction (blue) and the caudal tail angle (red) during a turn. The fish first swings its head toward the turning side with an amplitude of ΔΘ_H1_, which is followed by three undulations. The final heading direction (ΔΘ_H_ = 81°) is markedly different from the initial direction (0°), indicating the performance of a turn. (E) Histogram of heading direction changes concurrent with each swim. In response to the phototaxic visual stimuli, fish exhibit two modes of swimming behaviors: forward swims (ΔΘ_H_ ∼ 1.5°) and turns (ΔΘ_H_ ∼ 38.9°). Histograms in (E)–(J) were collected from the same 24 fish. (F) Histogram of the initial head swing angle. The forward swims are consistently initiated by a head movement toward the brighter visual environment (red arrowhead). (G) Analysis of tail movements during phototaxis, represented by a 2D histogram with tail deflection (Θ) plotted on the x axis and cycle period (P) on the y axis. A cycle-by-cycle analysis reveals that the two modes of behaviors differ in the first undulation cycle (left panel), but not in the later cycles (middle and right panels). (H–J) Head and tail kinematics during the optomotor response (OMR). The direction of the moving grating is constantly 90° away from the heading direction of the fish. See also Figure S1 and Movie S1.

To quantitatively induce turning behaviors of various amplitudes and tail beat frequencies, we identified three types of visual stimulation paradigms that are known to elicit robust and reliable responses: (1) a phototaxis-inducing illumination contrast consisting of uniform brightness on one side of the fish and darkness on the other, (2) an optomotor response (OMR)-inducing stimulus in which fish turn and swim to follow whole-field gratings moving in various directions, and (3) whole-field dark flashes that evoke large-angle turns. In the first two paradigms, the location and orientation of the visual stimulus were updated in real time such that visual input was spatially stable in the reference frame of the fish, regardless of the animal’s position and orientation (Figure 1A; see also Movie S1 available online). Swim kinematics such as the heading direction and the tail shape of larva were analyzed in real time at 500 frames/s (Figures 1B–1D and S1). In response to the phototaxic stimulus, larval zebrafish expressed two modes of behaviors: a forward swimming mode that exhibited little change in the heading direction (ΔΘ_H_ = 1.5°) that accompanied each swim, and a turning mode (ΔΘ_H_ = 38.9°) toward the illuminated side (Figure 1E). Although forward swims were associated with nearly no change in the final heading direction, the swims were consistently initiated by a head swing (ΔΘ_H1_) toward the illuminated side, indicating a biased initiation of forward swims (Figure 1F, arrowhead). In order to examine more closely how forward swims differ from turns, we analyzed tail undulations in a cycle-by-cycle manner. During the first cycle, both tail deflection (Θ_1_) and cycle period (P_1_) exhibited a bimodal distribution (Figure 1G, left panel). The forward swimming mode corresponded to a smaller tail deflection and a shorter cycle period (58° and 41 ms), whereas the turning mode corresponded to a larger tail bend and longer cycle period (143° and 59 ms). Interestingly, the bimodal distribution disappeared in the second undulation cycle (Figure 1G, middle panel). Later undulations between turns and forward swims were virtually identical (Θ_3_ = 59° and P_3_ = 45 ms; Figure 1G, right panel). Similar results were obtained with whole-field motion as the turn-inducing stimulus (Figures 1H–1J). Thus, despite the apparent difference between forward swims and turns, the two motor programs differed only during the first undulation cycle and were nearly identical in later undulations. Table 1 summarizes the swim kinematics during phototaxis, the OMR, the dark-flash response, and spontaneous swimming.

### Laser Ablation of Hindbrain vSPNs Affects the First Cycle of Tail Undulations and Promotes Forward Swims

The ventromedially located spinal projection neurons (vSPNs) consist of three bilateral pairs of nuclei, namely RoV3, MiV1, and MiV2, that are located at the ventromedial part of the hindbrain reticular formation [11, 14, 15]. These neurons are marked by the zebrafish homolog of mammalian Chx10 and provide glutamatergic innervation to the ipsilateral side of the spinal cord [16]. A previous study showed that vSPNs are necessary for the performance of turning behaviors induced by whole-field visual motion during the OMR [11]. There are at least three possible mechanisms that would explain these results. First, the vSPNs themselves might be capable of eliciting biased tail undulations, and forward swims might be controlled by an independent set of SPNs. In this case, removal of the vSPNs should lead to the absence of turning events without affecting forward swims. Second, the vSPNs might control individual tail flicks toward the left and right but also induce forward swims by becoming active simultaneously [17]. In this case, ablation would lead to a decrease in the occurrence of forward swims. A third possibility is that the cells might serve to switch forward swims, controlled by independent descending pathways, to turns by introducing an asymmetry to tail movements within a given swim event. In this case, ablation of the neurons would also predict a removal of turns but would result in an increase in the rate of forward swims.

**Figure 2:**
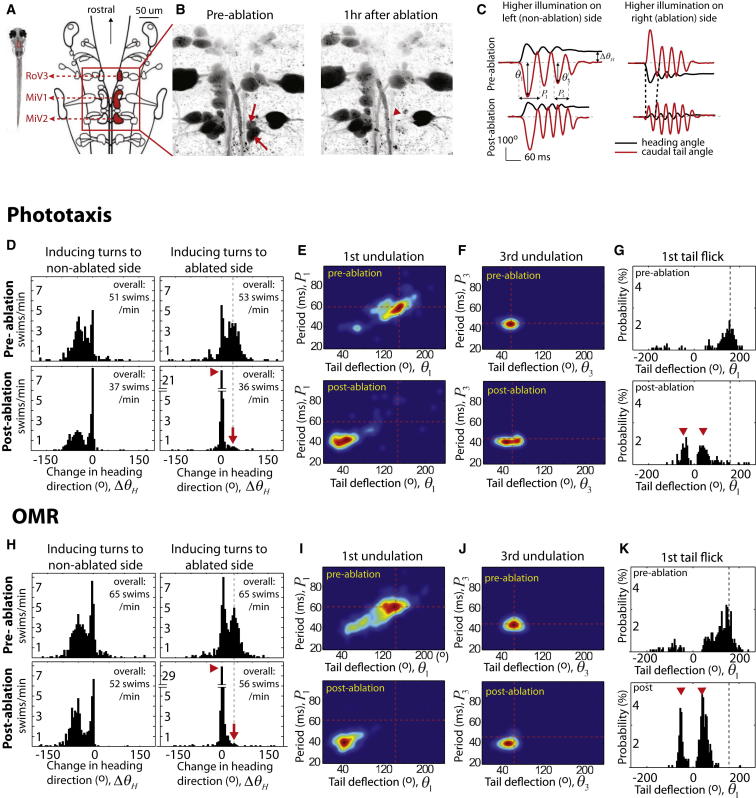
Laser Ablation of vSPNs Specifically Affects the First Undulation Cycle and Promotes Forward Swims during Phototaxis and the OMR (A) Schematic of hindbrain spinal projection neurons (SPNs) of larval zebrafish (image modified from [11]). (B) Two right MiV2 cells before and after laser ablation (arrows). The nearby ventral branch of the medial longitudinal fascicle (arrowhead) remains intact. (C) Example of ablation phenotypes. Visually induced right turns are replaced by forward swims after ablation of the vSPNs on the right (right panels). The amplitude of the first tail bend (Θ_1_) is weaker, and the period (P_1_) of the first undulation is reduced. Turning to the nonlesion side is unaffected (left panels). (D) Histograms of the change in heading direction (ΔΘ_H_) before and after vSPN ablation. The unilateral ablation abolishes turning to the lesioned side (red arrow) while drastically increasing the occurrence of forward swims (red arrowhead). Data were collected from the same eight fish to plot the histograms in (D)–(K). (E and F) Analysis of tail movements during phototaxis, represented by a 2D histogram with tail deflection (Θ) plotted on the x axis and cycle period (P) plotted on the y axis. The ablation affected the first undulation cycle (E), but not the later cycles (F). Dotted red line indicates the position of the preablation maximum. (G) Histograms of the angle of the initial tail bend. The amplitude of bends toward the lesioned side is greatly reduced after the ablation. Instead, small-angle bends on either side of the body are performed (red arrowheads). (H–K) During the OMR, the ablation also specifically affects the first undulation cycle of tail movements and promotes forward swims. See also Figure S2.

Here, we directly tested how hindbrain vSPNs modulate tail undulations during phototaxis, OMR, dark-flash response, and spontaneous swimming by ablating these neurons using a two-photon laser (Figures 2A–2C). We tested the behaviors of the same group of fish (n = 24) before and after ablation for all four visuomotor assays. Because the SPNs were labeled stochastically by spinal cord injections, the number of cells ablated and the consequent phenotype varied. However, we found that whenever one type of turn was abolished, the other three behaviors were also impaired. This indicates that these sensorimotor behaviors, which require the detection of spatial contrast, visual motion, and a temporal change in luminosity, use the same set of vSPNs in controlling turns.

In several cases (8 of 24 fish), where the ablation completely abolished turns toward the ablated side (Figure 2D, right panels), turns toward the contralateral side remained intact (Figure 2D, left panels). A cycle-by-cycle analysis of tail movements revealed that the fish still performed tail undulations after the ablation (Figure 2C, lower right traces). However, the first undulation cycle was severely affected by the ablation: the tail deflection and the cycle period dropped by 61% and 33%, respectively (Figure 2E). During the second undulation, the two parameters were reduced by only 6% and 2%. Later undulations were virtually unaffected by the ablation in terms of amplitude, period, and directional bias (Figure 2F). Similar ablation phenotypes were also observed in turning behaviors induced by visual motion (Figures 2H–2J). Overall, the ablation specifically abolished turning characteristics, namely large tail deflections and prolonged undulations, but spared the performance of symmetric tail undulations. These results show that vSPNs are not necessary for generating rhythmic tail undulations but instead might serve to transform a symmetric motor pattern into an asymmetric motion that underlies a turn. If this is true, removing the vSPNs should reveal the basic motor pattern of forward swims. This idea is supported by the observation that ablation not only abolished turns (phototaxis: from 34 ± 6.7 turns/min to 2.9 ± 2.1 turns/min, p = 0.001; OMR: from 34 ± 3.2 turns/min to 2.8 ± 1.5 turns/min, p = 0.000005) but also drastically increased the occurrence of forward swims (phototaxis: from 13 ± 3.8 swims/min to 31 ± 4.1 swims/min, p = 0.009; OMR: from 17 ± 5.2 swims/min to 49 ± 4.7 swims/min, p = 0.001; pooled data from the eight fish are shown in Figures 2D and 2H, arrowheads). The increase in the occurrence of forward swimming was striking, since we observed a general reduction in the overall swim frequency after the ablation (from 53 swims/min to 36 swims/min in phototaxis, and from 65 swims/min to 56 swims/min in OMR). Therefore, the increase in the probability of forward swims is likely due to the transformation of turns back into forward swims. Interestingly, the spared forward swims were initiated by tail bends toward either side of the body (Figures 2G and 2K, arrowheads), indicating that, unlike in vSPNs, the independent descending pathway that controls forward swims innervates both sides of the spinal circuitry. During the dark-flash response and spontaneous swimming, ablation of vSPN also impaired turns to the ablated side and drastically increased the occurrence of forward swimming (Figure S2). Together, these ablation experiments show that vSPNs have a universal role in controlling visually induced and spontaneous turns, and that they control turning behaviors by increasing the tail deflection and the cycle period during the first undulation cycle of tail movements.

### Activity of vSPNs during Turns of Different Amplitude

**Figure 3:**
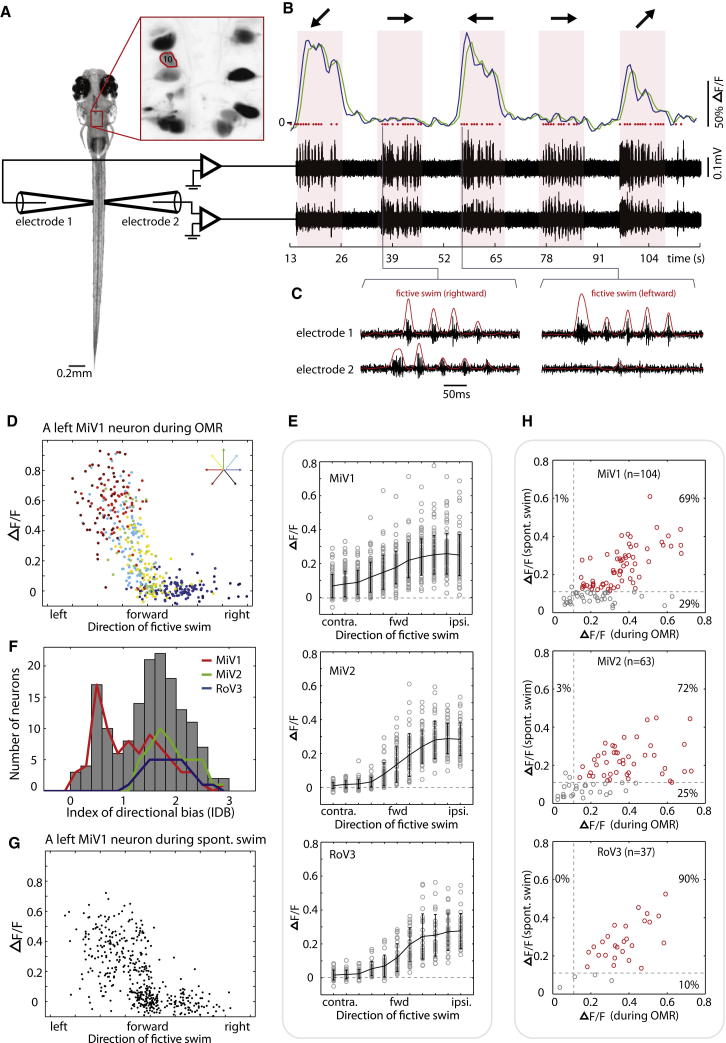
vSPNs Show Graded Responses with Respect to Turn Angle and Show Correlated Activity for Visually Evoked and Spontaneously Occurring Swim Events (A) Simultaneous recordings of motor nerve signals and hindbrain neuron activity reported by calcium imaging. (B) A left MiV1 cell (marked “10” in inset of A) backfilled with calcium green dextran responds strongly to left and backward left gratings and weakly to a forward right grating. Region-of-interest averaged fluorescence time series is shown in green, and the deconvolved trace is shown in blue. Motor nerve activity (black traces) was recorded bilaterally to identify fictive swims (red dots). (C) Motor nerve signals shown at higher resolution. Examples of a right turn (left panel) and a left turn (right panel) are shown. (D) Fluorescent calcium response (ΔF/F) of a MiV1 neuron as a function of swimming direction. Each dot represents a discrete swimming event, and the color indicates the visual stimulus used to elicit the swim. The cell exhibits a rectifying-shaped activation profile. (E) Activation profile of the three nuclei (83, 47, and 29 neurons in MiV1, MiV2, and RoV3 nuclei, respectively). Swim direction was divided into 11 bins, and each circle indicates the average ΔF/F of a neuron in the swim direction. Error bars represent SD. Only responsive neurons are analyzed (ΔF/F > 0.12; MiV1, 83 of 104 cells; MiV2, 47 of 63 cells; RoV3, 29 of 37 cells). (F) Analysis of the directional bias of all vSPNs reveals two functional groups. (G) Calcium responses of the same neuron shown in (D) during spontaneous fictive swimming. The same rectifying shape is apparent. (H) The majority of the vSPNs that are active during the OMR are also active during spontaneous swimming. Each circle represents a neuron. A threshold of ΔF/F > 0.12 (dashed lines) is used to define active cells. Neurons above thresholds are shown in red; neurons below thresholds are shown in gray. See also Figures S3 and S4.

The vigor of motor output can be controlled by the graded activity of the same set of neurons [18], or by selective activation of different subsets of neuronal populations [19, 20]. We next set out to test how vSPNs encode a wide range of turn angles by correlating the calcium fluorescence of vSPNs with the turning strength of fictive swims (Figures 3A and 3B). In the fictive swimming paradigm [21, 22], the periodic bursting of peripheral motor nerves is recorded as a proxy for intended swims of the fish. These bursts occur every ∼40 ms for three to six repetitions with left-right alternations (Figure 3C), reminiscent of tail undulations during free swimming. The turning direction and strength were estimated by comparing the power difference between left and right motor nerve signals (see Supplemental Experimental Procedures). Using gratings moving in different directions, we elicited fictive swims covering a wide spectrum of turning angles (Figure S3). We examined 204 vSPNs from 20 fish and found that 159 neurons were active during fictive swims (29 of 37 cells in RoV3, 83 of 104 cells in MiV1, and 47 of 63 cells in MiV2). By correlating an individual neuron’s calcium activity to the intended swimming direction of the fish, we found that the majority of these vSPNs exhibited an activation profile of a rectifying or sigmoidal shape; they were silent during turns toward the contralateral side and weakly active during forward swims, and their activity progressively increased with the turning strength to the ipsilateral side (see Figure 3D for an example neuron). We quantified this observation by using the index of directional bias (IDB), which is the difference between responses of the cell during ipsilateral and contralateral turns (Figure 3F). We found two functional groups within the vSPNs. The first group, which consisted of 76% of the vSPNs, showed a strong directional bias for ipsilateral turns. This group included all responsive RoV3 and MiV2 neurons (n = 29 and 47 cells, respectively) and 54% of the MiV1 neurons (45 of 83 cells). The other functional group exhibited only a weak directional bias for ipsilateral turns and was found exclusively in the MiV1 nucleus (Figure 3F). The MiV1 neurons with a weak directional bias tended to be active during all swimming directions, and a few of them (11 of 83 cells) had elevated activity during weak ipsilateral turns (see Figure S4A for example). Thus, the overall population response differed among the three nuclei: RoV3 and MiV2 nuclei showed a clear sigmoidal or rectifying profile in their activation during swims, whereas the MiV1 nucleus was active during all directions, with a weak directional bias for ipsilateral turns (Figure 3E). We did not observe a continuous shift in the subset of vSPNs tuned for specific turning angles, suggesting that the strength of turns is not controlled by recruiting different subsets of neurons. Instead, the rectifying shape of the activation profile of most of these neurons makes it likely that the strength of turns is controlled by the same set of vSPNs in an activity-dependent manner.

The ablation experiments show that whenever a vSPN ablation leads to impairment in visually induced turns, spontaneous turns are also impaired. This suggests that the same subset of vSPNs is used to control both visually induced and spontaneous turns. Here, we directly examined whether neurons that are active during the OMR are also recruited during spontaneous swims by monitoring the calcium fluorescence of individual neurons (see Figure 3G for an example neuron). This can only be done by simultaneously recording motor nerve signals, because there is no visual stimulus to determine the onset of motor events during spontaneous swims. We found a high degree of overlap between neurons that were active during the visually evoked and spontaneously occurring swims. More than 69% of the responsive vSPNs were active in both behaviors (Figure 3H, red cells).

## Discussion

To study how SPNs in the brainstem generate descending motor commands, we compared detailed kinematics between forward swims and turns, and we found that the two apparently distinct motor outputs differed only during the first undulation of tail movements. Removal of a discrete subset of ventromedial hindbrain SPNs, namely RoV3, MiV1, and MiV2 neurons, specifically abolished turning kinematics during the first undulation cycle, while symmetric tail undulations throughout the swim were spared. This cycle-specific modulation of behaviors by the vSPNs occurred during various types of visually elicited turns as well as during spontaneous locomotion. The activation profile of the vSPNs further supports the notion that these neurons encode turning angles of different sizes via a change in their activity levels.

### Modular Design of Descending Motor Control

Turn-controlling SPNs can generate biased swims in one of two ways: they can either (1) generate a template for a complete waveform of tail movements that exhibit directional bias or (2) modulate an independently generated symmetric motor output such that the combined output is a biased tail movement. Our results support the latter scenario. First, turns and forward swims differed only in the first undulation cycle, suggesting that a moderate modulation is sufficient to transform one behavior to the other. Second, symmetric tail undulations were spared after the ablation of vSPNs, indicating that the neurons do not serve to generate rhythmic body movements. Third, the probability of forward swims dramatically increased after the ablation of vSPNs, strongly suggesting that turns are transformed to forward swims in the absence of vSPNs. Together, these observations suggest that turning behaviors are generated by a concerted action of two groups of SPNs: one that generates symmetric, rhythmic body movements that result in a forward swim, and another, mediated by vSPNs, that introduces a biased, prolonged tail deflection during the first cycle of tail movements. The SPNs that elicit forward swims are currently unknown. However, neurons that are active during visually elicited forward swims are potential candidates. In larval zebrafish, these SPNs are present in various locations, including the nucleus of the medial longitudinal fasciculus (nucMLF) in the midbrain, the RoL1 nucleus in the hindbrain, and identified hindbrain neurons such as RoR1 and RoM1c [11]. The concerted action of SPNs in generating behaviors is reminiscent of modular designs. A modular system can be subdivided into smaller parts (modules) that are responsible for discrete functions. Each module is independent, and thus the deletion of one module will not affect the operation of others. Furthermore, different combinations of modules allow the system to express different functions. Here, we show that removing vSPNs spares the expression of symmetric tail undulations. Previous studies also showed that removing Mauthner cells and their homologs, while drastically increasing the response delay of escape turns, spared the expression of a wide spectrum of turning angles [6, 9]. It appears that different populations of SPNs specifically control different aspects of locomotor behaviors, and combined activations of these populations would generate various types of behaviors. For example, activation of vSPNs during symmetric tail undulations would result in turning behaviors, and an additional activation of the Mauthner neuron and its homologs would further shorten the response delay and result in high-performance escape turns. The function of the rest of the SPNs remains to be identified, but it is likely that they serve to introduce additional properties into the locomotor repertoire.

### Potential Modulation of Spinal Central Pattern Generators by vSPNs

The vSPNs provide descending excitation to the ipsilateral side of the spinal network [14–16]. Our ablation experiments show that vSPNs serve to increase the tail deflection and the cycle period during the first cycle of tail undulations. To increase tail deflections, vSPNs may simply innervate spinal motor neurons on the ipsilateral side. Controlling the undulation frequency, however, may involve more intricate regulation of the central pattern generator (CPG) network, since a prolonged tail bend requires an extended inhibition to the contralateral side of the spinal circuitry. Indeed, several lines of evidence obtained in lampreys suggest that one of the roles of commissural inhibition is to slow down the rhythm of the spinal network [23, 24]. In larval zebrafish, several commissural interneuron subtypes have been identified [25–27] that might serve as putative targets for descending vSPNs, and their modulation might explain the specific frequency changes that occur when a forward swim gets switched into a turn. Clearly, further experiments to describe the specific connectivity between SPNs and spinal neurons, and the combination of anatomical data with modeling studies, are needed to resolve these issues.

In summary, we have provided a clear example of how a specific locomotor behavior can be switched into a distinctly different behavioral event by the activation of a small set of SPNs. These SPNs generate new behaviors by introducing alternate kinematics into a basic motor pattern, such as transforming forward swims into turns. The independent control of turning kinematics and symmetric undulations suggests that a modular design is used in the central brain to construct descending motor commands that are sent into the spinal cord to generate behavior.

## Figures and Tables

**Table 1:** Comparison of Turning Kinematics during Different Visual Environments

	Change in Heading Direction (ΔΘ_H_) (°)	Maximal Change in Heading Direction (ΔΘ_H1_) (°)	Angle of 1^st^ Tail Bend (Θ_1_) (°)	Time to First Bend (ms)	1^st^ Cycle Period (P_1_) (ms)	Angle of 2^nd^ Tail Bend (Θ_2_) (°)	2^nd^ Cycle Period (P_2_) (ms)	Angle of Later Tail Bends (Θ_3_) (°)	Later Cycle Period (P_3_) (ms)	% of Swims with 3–6 Undulations
Spontaneous forward swims (n = 1,385)	0.4 ± 0.1	11.3 ± 0.2	54.5 ± 0.4	10.1 ± 0.1	40.3. ± 0.1	69.4 ± 0.3	39.7 ± 0.1	56.5 ± 0.2	43.6 ± 0.04	97.8%
Spontaneous turns (n = 3,104)	21 ± 0.4	39.3 ± 0.4	116.4 ± 0.6	17.9 ± 0.1	52.7 ± 0.2					
Turns in OMR (n = 4,458)	39.1 ± 0.3	56.1 ± 0.3	134.0 ± 0.4	19.6 ± 0.1	56.2 ± 0.2	76.5 ± 0.2	40.0 ± 0.02	61.8 ± 0.1	43.7 ± 0.03	96.5%
Turns in phototaxis (n = 3,704)	38.9 ± 0.4	61.0 ± 0.4	142.9 ± 0.5	20.6 ± 0.1	59.3 ± 0.2	73.2 ± 0.2	40.9 ± 0.1	58.8 ± 0.2	44.6.0 ± 0.03	97.7%
Turns in dark-flash response (n = 248)	123.0 ± 2.7	166.2 ± 1.9	227.4 ± 1.5	26.4 ± 0.4	74.5 ± 0.8	76.4 ± 1.2	40.6 ± 0.3	60.7 ± 0.8	46.7 ± 0.1	97.7%

The forward swimming mode during spontaneous swims is included as a reference of a nonturning pattern. Starting from the second undulation cycle, the forward swimming mode and turning mode are indistinguishable, and the data are pooled. See Figure 1D for symbol illustrations.

## Supplemental Information

Document S1. Figures S1–S4 and Supplemental Experimental Procedures

Movie S1. Phototaxis Assay and Analysis of Swim Kinematics, Related to Figure 1 A freely swimming larval zebrafish is exposed to a visual stimulus that is dark on the right side and bright on the left side. The stimulus is locked to the location and orientation of the fish. The consistent turns of the animal toward the bright side are apparent. Movie is shown in real time. The second part of the movie illustrates the details of the tracking algorithm; this section is slowed down 20×.
