# Supplementary material for: Spinal Projection Neurons Control Turning Behaviors in Zebrafish
Source: Curr Biol. 2013 Aug 19;23(16):1566–73. doi: 10.1016/j.cub.2013.06.044 (PMC3752323; doi:10.1016/j.cub.2013.06.044)
Supplement: Document S1. Figures S1–S4 and Supplemental Experimental Procedures [file mmc1.pdf]

Current Biology, Volume *23*

Supplemental Information

Spinal Projection Neurons Control

Turning Behaviors in Zebrafish

Kuo-Hua Huang, Misha B. Ahrens, Timothy W. Dunn, and Florian Engert

Supplemental Inventory

Figure S1. Tail tracking algorithm

Figure S2. Laser ablation of vSPNs abolishes turns during spontaneous swimming and the dark flash response

Figure S3. Histogram of the directional bias of fictive swims in response to moving gratings

Figure S4. Additional examples of activation profiles of vSPNs

Supplemental Experimental Procedures

Supplemental References

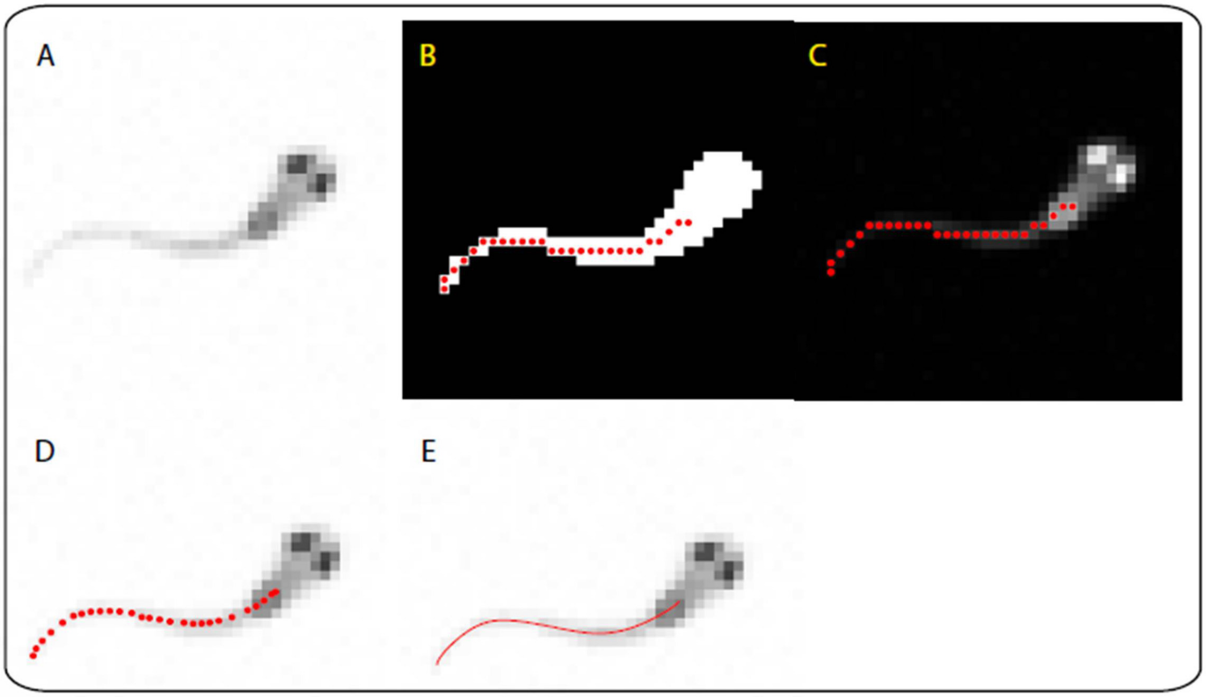

Figure S1. Tail Tracking Algorithm

(A) A grayscale image of larval zebrafish after background subtraction.

(B) The grayscale image is thresholded and undergoes a thinning process using MATLAB function *bwmorph*. The integer coordinates (red dots) generated by the thinning process lead to either rigid lines or unnatural zigzags.

(C) An inverted image of (A) overlaid by the integer coordinates.

(D) Each integer coordinate is adjusted based on the intensity of the surrounding pixels. The adjusted coordinates faithfully track the midline of the fish in the image.

(E) Result of cubic splines connecting the adjusted coordinates.

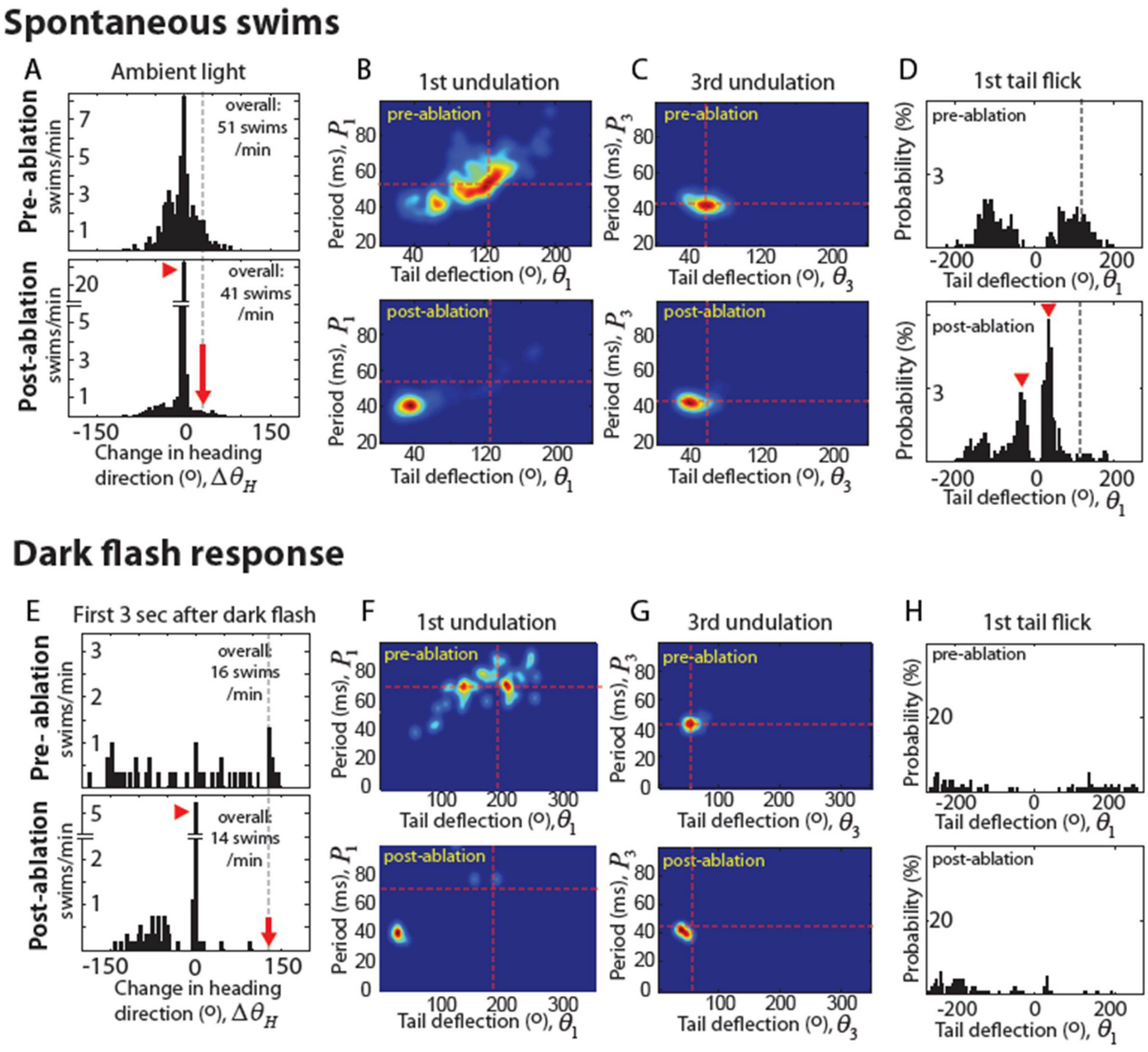

Figure S2. Laser Ablation of vSPNs Abolishes Turns during Spontaneous Swimming and the Dark Flash Response

(A) Histogram of the change in heading direction ($\Delta\Theta_H$) during spontaneous swimming. After ablation, turning to the ablated side is abolished (lower panel, red arrow) while the occurrence of forward swimming drastically increases (red arrowhead). The ablation phenotype is manifested more clearly in tail movements (B and D).

(B) Analysis of tail movements shows that the ablation mainly affects the first undulation cycle by reducing the tail bend angle (Θ_1) and the cycle period (P_1).

(C) Later undulation cycles are virtually unaffected by the removal of the spinal projection neurons.

(D) Histograms of the angle of the initial tail bend.

(E) A previous study has described a type of large-angle turns induced by a sudden reduction of illumination, namely a dark flash. Unlike touch-elicited escape turns, the turns induced by a dark

flash have a long response delay (mode 1500ms vs. 8ms) and are Mauthner cell independent [1]. We tested whether this behavior is mediated by the vSPNs. Before the ablation, a sudden dark flash induces a wide spectrum of turning angles, some of which results in a heading-angle change ($\Delta\Theta_H$) of more than 125°. After the ablation, the visual stimulus mostly induces turns toward the non-ablated side as well as forward swims (red arrowhead). Turning toward the ablated side rarely occurs (red arrow).

(F-H) Analysis of tail movements during the dark flash response.

All data were collected from the same 8 fish shown in Figure 2.

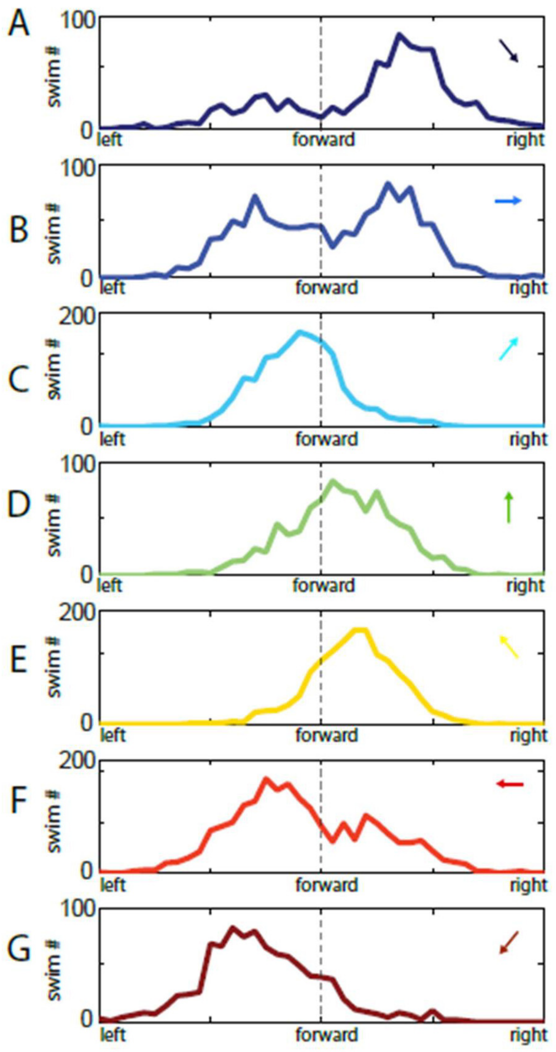

Figure S3. Histogram of the Directional Bias of Fictive Swims in Response to Moving Gratings

The intended swimming direction of a paralyzed fish is calculated by comparing the power of motor nerve signal on two sides of the tail.

(A and G) Gratings moving to backward right (135°) and backward left (-135°) induce fictive swims with large directional bias following the direction of the stimulus.

(B and C) Gratings moving laterally (90° or -90°) mainly induce swims toward side where the stimuli are moving to, but sometimes induce swims toward the opposite direction.

(C and F) Forward-lateral moving gratings (45° or -45°) induce swims with small directional bias, but the bias is often on the contralateral side of the grating direction.

(D) Forward moving gratings (0°) induce swims with directional bias centered near zero.

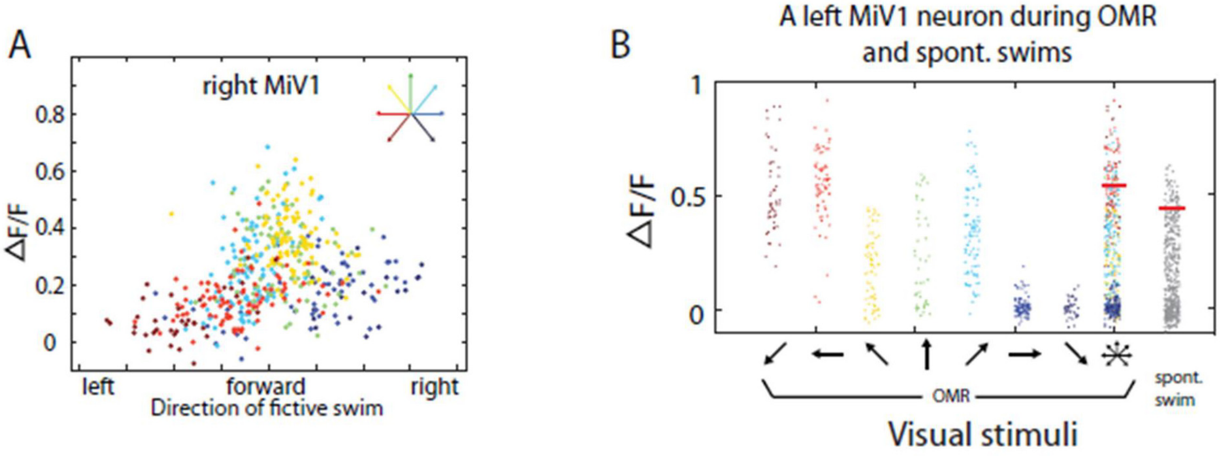

Figure S4. Additional Examples of Activation Profiles of vSPNs

(A) Example of the activation profile of a MiV1 neuron that exhibits a weak directional bias. Instead of exhibiting a rectifying activation profile as shown in Figure 3D, a subset of MiV1 neurons (11 out of 83 cells) exhibits an elevated activity during weak ipsilateral turns. Each dot indicates the calcium fluorescence of the cell during a fictive swim and the color indicates the visual stimulus used to elicit the swim.

(B) Comparing a neuron's activity during the OMR and spontaneous swims. To analyze whether the vSPNs that were active during OMR were also active during spontaneous swims, for each cell we sorted the swim-triggered calcium fluorescence (ΔF/F) from low to high during OMR or spontaneous swimming, and used the 90th percentile of this rank (red bars) to indicate how active the cell was during each of these behaviors. Each dot indicates the calcium fluorescence of the cell during a swim under the influence of the visual stimuli.

Supplemental Experimental Procedures

Fish

6-8 day-old *mitfa-/-* (nacre) fish [2] were used in all experiments, as they lack pigment in the skin, but retain normal eye pigmentation. Their behavior is indistinguishable from wild-type siblings in the assays. Animal handling and experimental procedures were approved by the Harvard University Standing Committee on the Use of Animals in Research and Training.

Behavioral Assays and Laser Ablation

Locomotion of the larval zebrafish can be described in two ways: One is to measure the heading direction (Θ_H) of the fish, which is described by the direction of the perpendicular bisector of the line connecting the centers of the two eyes, or, more simply, the direction in which the fish's nose is pointing (Figure 1B, blue arrows). Since larval zebrafish swim in discrete bouts, each swim provides a discrete readout of the change in heading direction ($\Delta\Theta_H$, Figure 1D, blue trace). A forward swim will correspond to a zero or small directional change, and a turn corresponds to a large directional change. An alternative way to measure the locomotion of the fish is to describe the evolution of the shape of the tail. This can be achieved by calculating the tangent vectors along the tail (Figure 1B, orange arrows) and subtracting their angles from the angle of the heading direction (result see Figure 1C). Since the movement of the tail is more flexible than the head, this measurement provides a robust readout of behavioral kinematics. For example, the deflection angle (Θ) and cycle period (P) of the caudal tail reliably capture rhythmic body movements (red trace in Figure 1D, or the last row of Figure 1C).

36 hrs before the experiment, 3% (w/v) solution of dextran-conjugated Texas Red (Invitrogen) was injected into the spinal cord of tricaine-anesthetized fish to label spinal projection neurons. The behavior of the injected fish was indistinguishable from non-injected siblings at the day of experiments. Before and after ablation of spinal projection neurons using two-photon laser [3], spontaneous and visually-elicited behaviors of an individual fish freely swimming in a 10-cm diameter petri dish were examined. The swimming behavior was recorded at 500 frames per second using an infrared-sensitive CMOS camera (Mikrotron, MC1362). Visual stimuli were projected directly onto a screen 5 mm below the fish using a DLP projector (Optoma). The position and orientation of the fish was analyzed in real time using custom software written in LabVIEW.

Tail motion was analyzed using custom MATLAB code. The crude midline of the fish body in the image was calculated using the *bwmorph('thinning')* function. Each of the identified pixels deviated slightly from the true midline due to its integer coordinate (x, y). As a larval zebrafish in the image was represented by merely 40-by-4 pixels, these integer coordinates lead to a tracking result that is either rigidly straight or unnaturally zigzagged. The coordinate was thus adjusted to take on decimal precisions (x', y') by considering the brightness of the surrounding pixels such that

$$x' = x + \frac{\sum_{i=-N}^{N} i \cdot \sum_{j=-N}^{N} P(x+i, y+j)}{\sum_{i,j} P(x+i, y+j)}, \quad y' = y + \frac{\sum_{j=-N}^{N} j \cdot \sum_{i=-N}^{N} P(x+i, y+j)}{\sum_{i,j} P(x+i, y+j)},$$

where P is the pixel brightness and N determines the range of surrounding pixels which is usually set to 1. This adjustment effectively "drifts" the integer coordinates back to the true

midline where the pixels tend to be locally brightest in the inverted image (supplemental Figure S1). The adjusted tracking points were then interpolated by cubic spline. The algorithm smoothly tracks the shape of the tail during all types of swims including minute J bends [4] and extreme O bends [1].

During the phototaxis-, the OMR- and the dark flash response-assays, the visual stimulus was presented for 10 sec followed by a homogenous illumination for at least 10 sec. The next trial was only initiated when the fish was 2cm away from the wall of the petri dish in order to prevent biased turns caused by the physical barrier. Spontaneous swimming was recorded under homogenous illumination for 5 minutes. In most cases, fish could swim immediately after SPN ablation, and were tested for the ablation phenotype. Fish that couldn't swim or lost their balance were not included in the analysis. Turning and forward swimming angles in Table 1 were obtained by fitting two normal distributions to the histogram of the change in the heading direction (ex. Figure 1E &1H). To distinguish turns and forward swims, we used a threshold of $\Delta\Theta_H = 20°$, which was determined by the intersecting point of the two Gaussian curves. However, we used a more stringent criterion ($\Delta\Theta_H < 10°$) to define forward swimming when we analyzed the occurrence of forward swimming after laser ablation. This is to ensure that the observed increase in forward swimming is not confounded by the potential increase in weak turns.

Calcium Imaging

36 hrs before the experiment, 25% (w/v) solution of dextran-conjugated calcium green (Invitrogen) was injected into the spinal cord of tricaine-anesthetized fish. On the day of the experiment, the fish was embedded in 2% low-melting temperature agarose and paralyzed by injecting 1mg/ml bungarotoxin solution (Sigma-Aldrich) into tail muscles. After removing the agarose surrounding the tail, two glass electrodes were attached with suction to intersegmental boundaries around the 10th myotome, which is rostral to the injection sites of the bungarotoxin and the calcium fluorescent dye. Simultaneous calcium imaging and motor nerve recording were made as previously described [5]. Briefly, a custom built laser scanning two-photon microscope, utilizing a Mai Tai pulsed infrared laser (Spectra Physics) tuned to 920nm, was used to monitor the calcium fluorescent signal of the spinal projection neurons. Square gratings with spatial period of 1.2cm moving at1cm/s were projected onto a diffusive screen underneath the fish via a 3M MPro110 mini projector. The light source of the projector was replaced by a red Luxeon LED that was pulsed in synchrony with the fast scan mirror, such that the red photons would not corrupt the green fluorescent signal of the image. Gratings moving in seven different directions were presented in random orders; each presentation lasted 10 seconds and each grating direction was presented 10 times per imaging plane. Imaging planes were 2 microns apart, and a typical experiment covered a volume about 100-by-100-by-40 microns. In each experiment, frames were acquired at 1.7 Hz. Due to the fact that neurons in the RoV3, MiV1 and MiV2 nuclei are tightly packed, the image was analyzed by manually selecting regions of interest. The calcium time series of each cell (Figure 3B, green trace) was deconvolved (Figure 3B, blue trace) by a decaying exponential to remove the slow dynamics of the calcium indicator [6]. The exponential has a time constant of 1.2 sec, which was estimated from the decaying calcium time series after a response-evoking stimulus is turned off. The deconvolved fluorescence signal was correlated to the directional bias of each fictive swim to obtain the activation profile of the cell (e.g. Figure 3D).

To analyze whether the RoV3, MiV1 and MiV2 neurons that were active during the OMR were also active during spontaneous swims (Figure 3H), for each cell we sorted the swim-triggered calcium fluorescence (ΔF/F) from low to high during OMR or spontaneous swimming, and used the 90th percentile of this rank to indicate how active the cell was during each of these behaviors (Supplemental Figure S4B, red bars).

Fictive Swim Recordings and Analysis

Motor nerve recordings were made as previously described [5, 7]. Briefly, two glass electrodes (used with a Multiclamp 700M amplifier) were attached with suction to the tail, on intersegmental boundaries around the 10th myotome. Electrical signals representing fictive swims typically became apparent ten minutes after the placement of the electrodes. When a lateral moving grating is presented, the initial burst occurs on the side that would initiate a turn into the moving direction and it has a longer bursting duration and higher firing frequency (Figure 3C), presumably corresponding to a prolonged and stronger tail bend at the beginning of a turn (cf. Figures 1D). The signal was processed by taking a windowed standard deviation so that if $s(t)$ is the electrical signal and $\bar{s}(t)$ the processed signal, then $\bar{s}(t) = SD(s(t-\tau/2)...s(t+\tau/2))$, with τ = 10ms. The processed signal has a high signal-to-noise ratio, and represents the power of the original electric signal. After subtracting the DC component contributed by noise, the power difference between the left and right channels during the first 50ms of each fictive swim was used to calculate the directional bias. This time window usually covers the first undulation cycle of forward swims and reveals a near-zero directional bias. For turns, this calculation revealed a large directional bias since the bursting activity on the turning side was prolonged and enhanced. The relative strength of the left and right electrodes was calibrated every 30 min by equalizing the power of the two electrodes during swims induced by forward moving gratings. The neuron's activation profile is the cell's calcium fluorescent response (ΔF/F) as the function of fictive swimming direction (Figure 3D). The index of directional bias (IDB) is the difference of the neuron's response during strong ipsilateral turns and contralateral turns and normalized by the mean of the neuron's response. The response of strong turns is the average ΔF/F of the three most extreme bins.

Supplemental References

1. Burgess, H.A., and Granato, M. (2007). Modulation of locomotor activity in larval zebrafish during light adaptation. J. Exp. Biol. *210*, 2526-2539.
2. Lister, J.A., Robertson, C.P., Lepage, T., Johnson, S.L., and Raible, D.W. (1999). nacre encodes a zebrafish microphthalmia-related protein that regulates neural-crest-derived pigment cell fate. Development *126*, 3757-3767.
3. Orger, M.B., Kampff, A.R., Severi, K.E., Bollmann, J.H., and Engert, F. (2008). Control of visually guided behavior by distinct populations of spinal projection neurons. Nat. Neurosci. *11*, 327-333.
4. Budick, S.A., and O'Malley, D.M. (2000). Locomotor repertoire of the larval zebrafish: swimming, turning and prey capture. J. Exp. Biol. *203*, 2565-2579.
5. Ahrens, M.B., Li, J.M., Orger, M.B., Robson, D.N., Schier, A.F., Engert, F., and Portugues, R. (2012). Brain-wide neuronal dynamics during motor adaptation in zebrafish. Nature *485*, 471-477.
6. Miri, A., Daie, K., Arrenberg, A.B., Baier, H., Aksay, E., and Tank, D.W. (2011). Spatial gradients and multidimensional dynamics in a neural integrator circuit. Nat. Neurosci. *14*, 1150-1159.
7. Masino, M.A., and Fetcho, J.R. (2005). Fictive swimming motor patterns in wild type and mutant larval zebrafish. J. Neurophysiol. *93*, 3177-3188.
