# Supplementary figures and images for: Spinal Projection Neurons Control Turning Behaviors in Zebrafish
Source: Curr Biol. 2013 Aug 19;23(16):1566–73. doi: 10.1016/j.cub.2013.06.044 (PMC3752323; doi:10.1016/j.cub.2013.06.044)

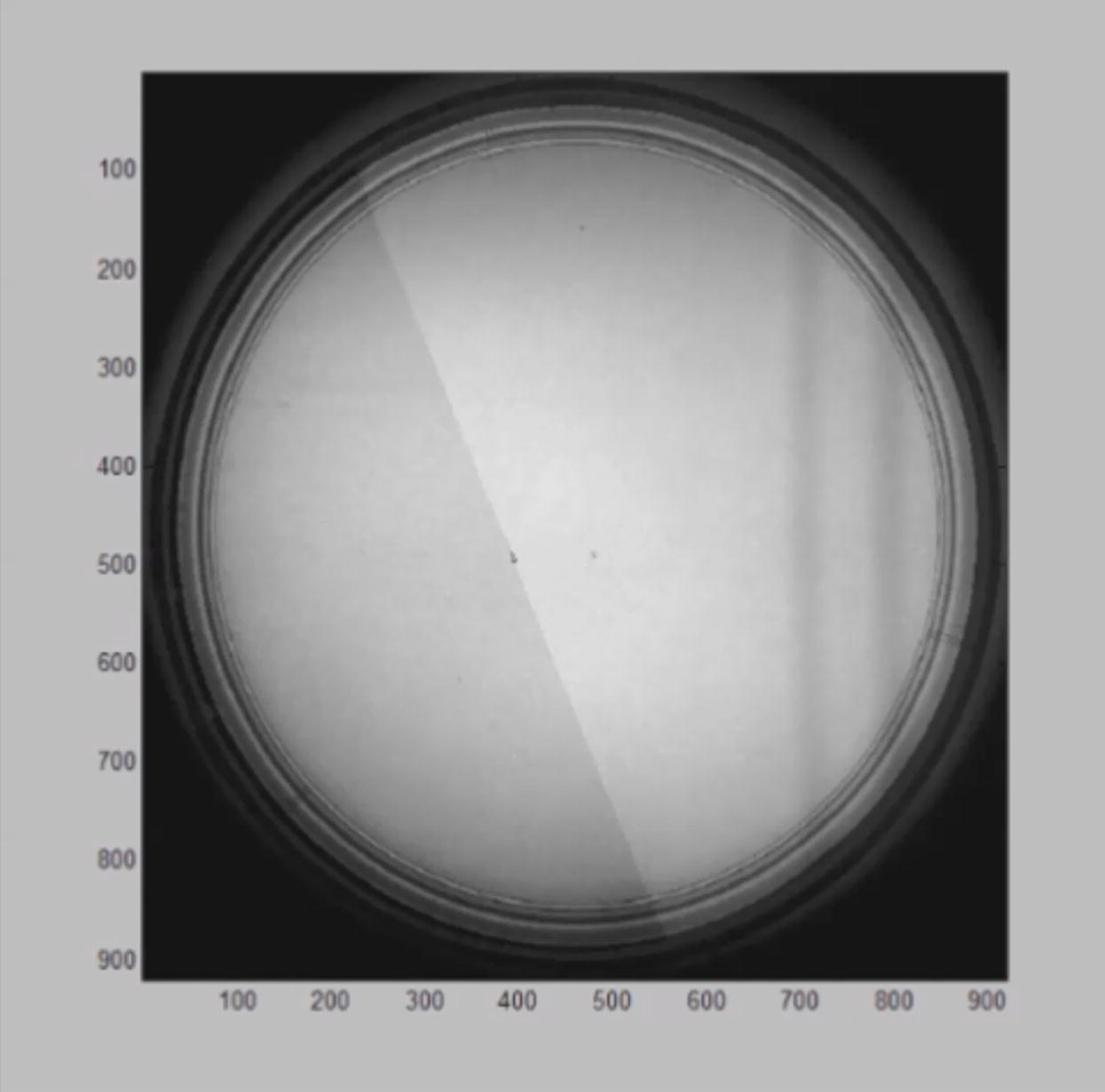

Supplement: Movie S1. Phototaxis Assay and Analysis of Swim Kinematics, Related to Figure 1 — A freely swimming larval zebrafish is exposed to a visual stimulus that is dark on the right side and bright on the left side. The stimulus is locked to the location and orientation of the fish. The consistent turns of the animal toward the bright side are apparent. Movie is shown in real time. The second part of the movie illustrates the details of the tracking algorithm; this section is slowed down 20×. [file mmc2.jpg]
